# Practical 3D Reconstruction and 3D Printing of Veterinary CT Scans in Small Animals: A Technical Demonstration with Reader-Based Validation in Canine Cranial Trauma

**DOI:** 10.3390/vetsci13070610

**Published:** 2026-06-24

**Authors:** Yuan Chai, Luxin Lou

**Affiliations:** 1 ORBIT Lab, College of Medicine and Biological Information Engineering, Northeastern University, Shenyang 110016, China; 2 The University of Sydney, Sydney Musculoskeletal Health and The Kolling Institute, Northern Clinical School, Faculty of Medicine and Health and the Northern Sydney Local Health District, Sydney, NSW 2065, Australia; 3 Radiology Department, Beijing Jishuitan Hospital, Capital Medical University, Beijing 100035, China; xxllxin@126.com

**Keywords:** computed tomography scan, 3D reconstruction, 3D printing, cranial fracture, neurosurgery

## Abstract

Traumatic fractures are common in small animal emergency care, yet subtle fracture lines may be difficult to identify accurately using routine three-dimensional reconstruction workflows, particularly when access to specialized software is limited. This study describes the use of the open-source platform Three-Dimensional Slicer for computed tomography-based reconstruction and three-dimensional printing in a small dog with cranial trauma, with emphasis on documenting a practical and reproducible workflow through voxel resampling. Imaging data were imported into the software, bone structures were segmented using a rapid workflow, voxel spacing was resampled for smoother surface visualization by volume resampling, and the reconstructed model was processed for physical printing. Digital models of different resolutions were generated within minutes, and a life-size skull model was successfully fabricated using fused deposition modeling in less than three hours at a material cost of under one United States dollar. The enhanced model provided an intuitive representation of fracture morphology and spatial relationships compared with routine reconstruction alone. These findings demonstrate that open-source software combined with low-cost printing can provide a rapid, affordable, and user-friendly approach for practical skeletal reconstruction in small animals, with practical value for fracture assessment, preoperative planning, and broader use in resource-limited veterinary settings.

## Simple Summary

Fractures caused by traffic accidents are common in small dogs and other companion animals, but fine fracture lines may be difficult to recognize from routine images alone. This study shows how a free software program can be used to efficiently turn CT scan data into three-dimensional digital models and physical printed models of injured bones. Using the skull of an injured small dog as an example, we describe a practical workflow for loading the scan, separating bone from surrounding tissue, resampling the volume for smoother surface visualization, and preparing the model for 3D printing. The digital models were created within minutes, and a life-size skull model was printed in less than three hours at a material cost of under one US dollar. The improved models provided a more intuitive view of the fracture pattern than routine reconstruction alone. This approach offers a fast, low-cost, and accessible way to support fracture assessment, communication, teaching, and selected planning tasks in veterinary practice, particularly in clinics that do not have access to expensive commercial software.

## 1. Introduction

Traumatic fractures are among the most common injuries encountered in small animal emergency care, particularly in densely populated urban environments where traffic accidents are frequent [1]. In a large VetCompass study of 199,464 dogs attending UK primary-care practices, road traffic accidents were reported in approximately 4 per 1000 dogs, with younger dogs being particularly affected and more than one-fifth of affected dogs dying from the event [2]. Timely and accurate identification of fractures is essential for effective surgical planning and favorable prognostic outcomes [3,4]. Computed tomography (CT) has emerged as a valuable diagnostic tool in veterinary medicine, offering high contrast resolution and the ability to visualize complex anatomical structures in three dimensions [1,5].

However, its utility in detecting subtle or fine fractures in small animals remains limited by practical and technical constraints [1]. The apparent clarity of derived three-dimensional surface models can be influenced by voxel spacing, image noise, partial-volume effects, reconstruction settings, and operator choices during segmentation [6]. For small animals such as Yorkshire Terriers, whose skeletal structures are both diminutive and delicate, fractures may occupy only a few voxels—resulting in blurred or indistinct cortical boundaries under standard clinical settings [3]. Furthermore, the urgency of trauma cases often necessitates rapid image interpretation, increasing the risk of missed diagnoses when fracture lines are subtle or obscured [7]. In clinical practice, commercially available 3D reconstruction software is typically expensive and often bundled with proprietary imaging hardware, posing economic and accessibility barriers for many veterinary clinics [7]. Consequently, practical open-source workflows for adjunctive visualization, education, communication, and preoperative planning remain relevant in general veterinary settings [8,9].

To address these challenges, this study proposes a practical, cost-free approach using 3D Slicer, an open-source software originally developed for human medical imaging research [10,11]. The “Resample Scalar Volume” module was used to generate a more isotropic voxel representation for surface rendering; this interpolation-based step does not increase the intrinsic spatial resolution of the original CT data or create new diagnostic information [5]. Accordingly, this study was designed as a technical demonstration of a rapid, low-cost, open-source workflow for CT-based 3D visualization and 3D printing in a small-animal cranial trauma case (Yorkshire Terrier), referred to as “Xiaoli” (nickname used in this study), who sustained multiple injuries following a traffic accident. We detail a complete workflow from CT data acquisition and voxel resampling to 3D modeling and physical printing. The novelty of this technical demonstration lies in presenting a simplified open-source CT-to-3D workflow for small-animal cranial trauma, combined with low-cost printing and exploratory reader-based assessment of visualization utility, with potential applications in surgical planning, education, and improved clinical outcomes.

## 2. Materials and Methods

### 2.1. Clinical Case and Imaging Dataset

Day 0 post-accident: An approximately 2-year-old male Yorkshire Terrier (2.2 kg; estimated maximum cranial coronal width ~50 mm) was found on the street with visible facial bleeding and was immediately transported to a veterinary hospital for emergency assessment. Whole-body anteroposterior and lateral radiographs revealed a cranial fracture localized to the left frontoparietal cranial vault region, with no additional osseous abnormalities identified in the axial or appendicular skeleton. Supportive care, including analgesia, hemostasis, and nutritional supplementation, was initiated. By post-injury day 11, the patient was assessed to be clinically stable for anesthesia, and an MRI was performed to assess intracranial and soft-tissue injury, whereas the present workflow focused on CT-based osseous segmentation because cortical bone is more directly and reproducibly reconstructed from CT data for 3D visualization and printing [12]. Cross-sectional imaging revealed an extensive scalp hematoma involving the frontal and parietal regions, accompanied by depressed calvarial fractures involving the frontal and parietal bones. The left cerebral hemisphere demonstrated heterogeneous signal intensity, loss of normal anatomical architecture, and a large intraparenchymal fluid signal—findings consistent with cerebral contusion. Surgical intervention was performed two days following imaging to evacuate hematoma-associated blood clots, expose the depressed fracture and partial orbital rim, remove displaced bone fragments, and repair the dura mater. The patient was discharged eight days postoperatively.

### 2.2. CT Imaging Acquisition

CT imaging was performed using a clinical scanner (Liao Ning Kampo Medical Systems Co., Ltd., Benxi, Liaoning, China; model: DBC Numen CT S; software: Polaris V2.2) as part of the patient’s trauma evaluation. The exported DICOM metadata recorded a 512 × 512 matrix, 0.8 mm slice thickness, 0.580 × 0.580 mm in-plane pixel spacing, 110 kVp, 32 mA tube current, and 11-s exposure time. Bone-window display settings were 400/2000 for window center/window width. This dataset should be interpreted as an available clinical CT dataset rather than an optimized micro-CT or research-grade high-resolution protocol. Potential effects of noise, partial-volume averaging, field of view, and reconstruction settings on visualization of small cranial structures are acknowledged. A total of 250 CT slices were acquired, resulting in a dataset size of approximately 125 MB.

### 2.3. Fast 3D Reconstruction

**Figure 1:**
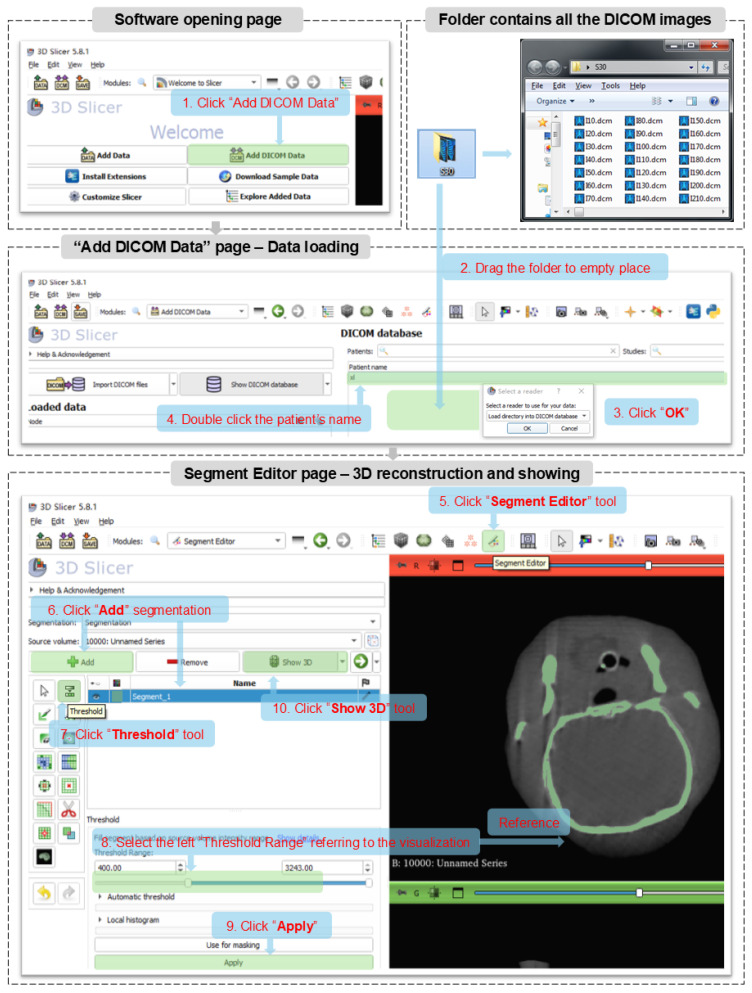
Rapid 3D reconstruction workflow in 3D Slicer. The panels illustrate the sequential steps for importing DICOM CT data, creating a new bone segmentation, applying threshold-based segmentation, and activating three-dimensional visualization of the segmented cranial bone structures.

To enable fast, intuitive, and clinically useful 3D reconstructions of skeletal structures, we employed 3D Slicer (version 5.8.1, https://www.slicer.org/, accessed on 1 April 2025), a free and open-source medical imaging software. Basic threshold-based 3D visualization can be generated within one minute after data loading, whereas additional voxel resampling, ROI cleaning, and preparation of printable models require an additional 5–20 min depending on the intended output. (Figure 1):

1. Loading DICOM Data (Steps 1–4): All DICOM files were first downloaded into a single folder (named “S30” in our case). In 3D Slicer, the “Add DICOM Data” module was opened. The folder was then dragged into the Slicer workspace. Upon confirming the prompt by clicking “OK” and double-clicking the appropriate image series (or patient name if only one series is present), the CT dataset was loaded successfully.
2. Bone Segmentation (Steps 5–9): The “Segment Editor” module was selected from the top menu. A new segmentation was created using the “Add” button. The “Threshold” effect was then applied to isolate osseous structures. The threshold values were adjusted manually by observing the highlighted regions in the axial, sagittal, and coronal planes—typically starting from the left-hand slider to exclude soft tissues while preserving as much of the high-intensity bone signal as possible. After fine-tuning, the threshold was confirmed by clicking “Apply.”
3. 3D Visualization (Step 10): Finally, the “Show 3D” button was activated to render a real-time 3D model of the segmented bones. This allowed for immediate 3D visualization of anatomical details, including cortical continuity, displacements, and potential fractures.

### 2.4. Voxel Resampling for 3D Surface Visualization

**Figure 2:**
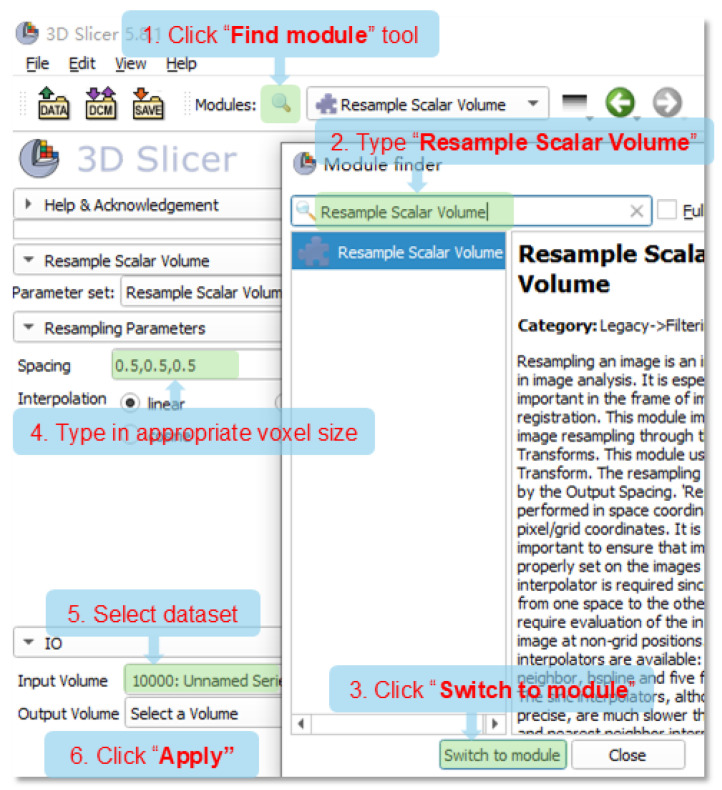
Voxel resampling workflow using the Resample Scalar Volume module in 3D Slicer. The panels show how the original CT voxel spacing is checked from DICOM metadata, how a target isotropic spacing is entered, and how the resampled volume is generated before subsequent segmentation and 3D visualization.

To generate a more isotropic voxel representation for surface rendering and 3D visualization, we employed the ‘Resample Scalar Volume’ module in 3D Slicer. This interpolation-based step does not increase the intrinsic spatial resolution of the original CT acquisition, but may improve the smoothness and consistency of the resulting 3D surface model. After completing step 4 in Figure 1 (loading the source data), refer to Figure 2: use the “Find module” tool to locate and open the “Resample Scalar Volume” module (steps 1–3). Next, input an appropriate “Spacing” value (step 4, see explanation below). Select the dataset to be processed, then click “Apply” (steps 5–6). After resampling in Figure 2, proceed with the segmentation process from step 5 of Figure 1, as previously described.

To determine suitable ‘Spacing’ settings in step 4 of Figure 2, values equal to or slightly smaller than the original voxel dimensions may be selected to obtain a more isotropic dataset for surface rendering. However, overly small spacing values increase processing time and do not create new diagnostic information or proportionally improve visualization quality. Operators can find the original voxel dimensions in the DICOM metadata: (1) Pixel Spacing (0028,0030): indicates the physical distance between the centers of adjacent pixels in the x (row) and y (column) directions, typically in millimeters; (2) Slice Thickness (0018,0050): represents the nominal thickness of each slice in the z-direction. For example, in our case, the “Pixel Spacing” value of “0.58\0.58” with a “Slice Thickness” of “0.8” denotes 0.58 mm spacing in both directions with slice intervals of 0.8 mm, corresponding to spacing value: 0.58,0.58,0.8. Accordingly, an appropriate “Spacing” value setting could be “0.5, 0.5, 0.5”.

### 2.5. Preparing 3D Printable Models

**Figure 3:**
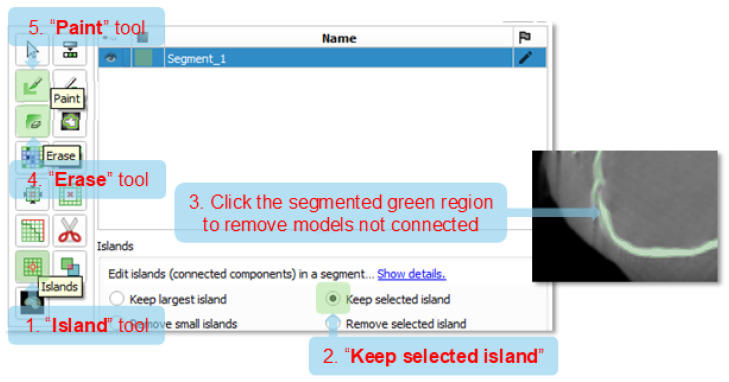
Post-processing steps to prepare segmented CT data into 3D-printable models.

3D printed physical models can be valuable for preoperative planning and intraoperative visualization. After generating the segmented 3D digital model (following step 10 in Figure 1), the output may not be immediately suitable for 3D printing due to the presence of numerous small, unconnected fragments. These typically arise from minor structures or noise captured during thresholding, whereas the printing objective is often to reproduce only the region of interest (ROI), focusing on bone surfaces rather than internal cavities or surrounding tissues. In this paper, we recommend using three segment editing tools to refine the model prior to export (Figure 3):

1. “Island” Tool—Use the “Island” tool and select “Keep selected island” to retain only the largest connected component of the ROI, effectively removing smaller, unconnected fragments (steps 1–3).
2. “Erase” Tool—Apply the “Erase” tool to manually remove anatomical regions outside the ROI (step 4). This step is especially important since larger models significantly increase 3D printing time and material consumption.
3. “Paint” Tool—To optimize the model for printing, especially when only the external bone surface is of interest, the “Paint” tool can be used to fill internal cavities (e.g., trabecular bone or brain spaces) with solid material. This solidification simplifies geometry, reduces surface complexity, and can accelerate the 3D printing process by minimizing internal surface area.

### 2.6. 3D Printing

The cleaned and refined model can then be exported as a .stl file for 3D printing, which can be achieved using the ‘Export to files’ function located next to the ‘Show 3D’ button. Depending on the specific printer and slicing software used, it is advisable to orient the model with a large, flat surface facing downward to minimize the need for support structures during printing. In many cases, the region of clinical interest—typically the bone surface—is oriented upward to ensure better surface quality and detail preservation without interference from support materials. This orientation should be planned during the model preparation stage. Additionally, the model can be scaled during the printing process: enlarging the model can aid in visualizing fine fracture lines, while maintaining the original scale is recommended for accurate preoperative templating and surgical planning [13,14].

### 2.7. Retrospective Reader-Based Assessment of the Added Value of 3D Visualization

An exploratory retrospective reader-based assessment was performed to evaluate the perceived complementary value of 3D visualization following conventional 2D CT review. Five independent evaluators with experience in veterinary imaging or musculoskeletal imaging completed two sequential assessment rounds.

In the 2D round, evaluators reviewed anonymized 2D CT materials consisting of five representative images in each of the axial, sagittal, and coronal planes, resulting in 15 images compiled into a PDF file. These images were selected by an independent veterinary radiologist who was not involved in the present study. No diagnostic annotations, radiology report, operative findings, or study conclusions were provided.

After a 5-day washout interval, the same evaluators completed the 3D round, in which they reviewed 3D visualization materials only. These included six standardized views of the reconstructed cranial model and a rotating GIF generated from the STL model. The GIF format was used to allow review on standard computers without dedicated 3D-viewing software.

After each round, evaluators completed an anonymous online questionnaire (Supplementary File S1) assessing fracture presence, location, and morphology, together with six five-point Likert-scale items: fracture-line visibility, fracture morphology understanding, spatial anatomical understanding, diagnostic confidence, surgical planning value, and overall clinical usefulness. In the 3D round, additional items assessed the perceived added value and limitations of the 3D materials. A score of 1 indicated very poor visualization or very low confidence, whereas a score of 5 indicated excellent visualization or high confidence. Questionnaire submission indicated consent for anonymized data use in scientific analysis and publication.

Given the single-case design and small sample size, results were analyzed descriptively. Scores are reported as median and interquartile range (IQR). A composite clinical utility score was calculated by summing the six Likert-scale items, with a maximum score of 30. Paired changes in reader-rated scores between the two sequential rounds were examined descriptively, with an exploratory Wilcoxon signed-rank test performed for the composite score.

## 3. Results

**Figure 4:**
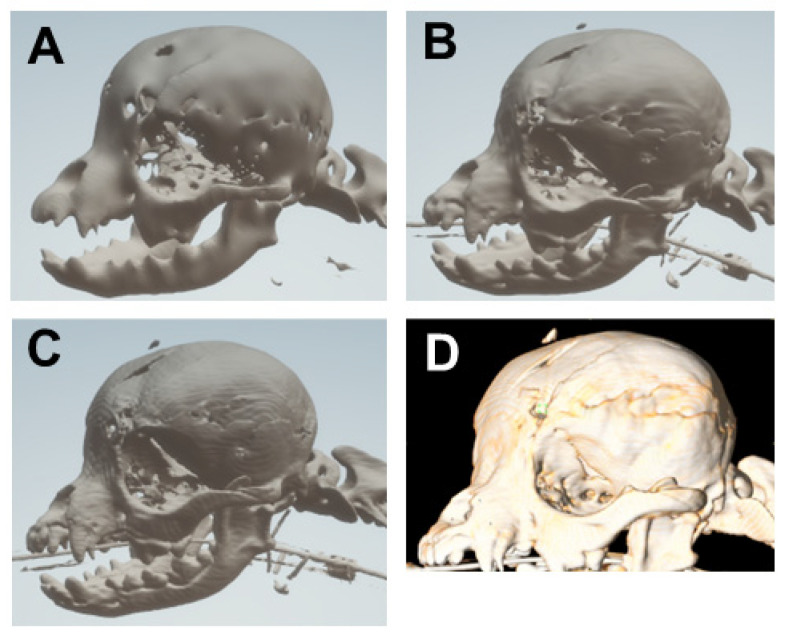
Comparison of 3D segmentation results generated from different voxel-resampling settings. (**A**) 1.0 × 1.0 × 0.8 mm^3^. (**B**) 0.5 × 0.5 × 0.5 mm^3^. (**C**) 0.25 × 0.25 × 0.25 mm^3^. (**D**) Standard clinical 3D reconstruction from the radiology report, generated from the original CT dataset with 0.58 × 0.58 × 0.8 mm^3^, without additional voxel resampling.

This study reconstructed the Yorkshire Terrier’s skull in three spatial resolutions using the same threshold range (Figure 4): the default resolution (voxel size 1.0 × 1.0 × 0.8 mm^3^, Figure 4A) and two enhanced resolutions with resampled voxel volumes of 0.5^3^ mm^3^ and 0.25^3^ mm^3^ (Figure 4B and 4C, respectively). A commercially rendered 3D screenshot from the radiology report is shown for comparison (Figure 4D). All models were prepared by a senior orthopedic engineer. Figure 4A was generated within 1 min using threshold-based segmentation immediately after data loading. Incorporating a resampling step to produce the higher-resolution model shown in Figure 4B took approximately 3 min. These steps were executed on a mid-range laptop (CPU 11th Gen Intel i7-1165G7, GPU NVIDIA MX450 2 GB, RAM 16 GB).

**Figure 5:**
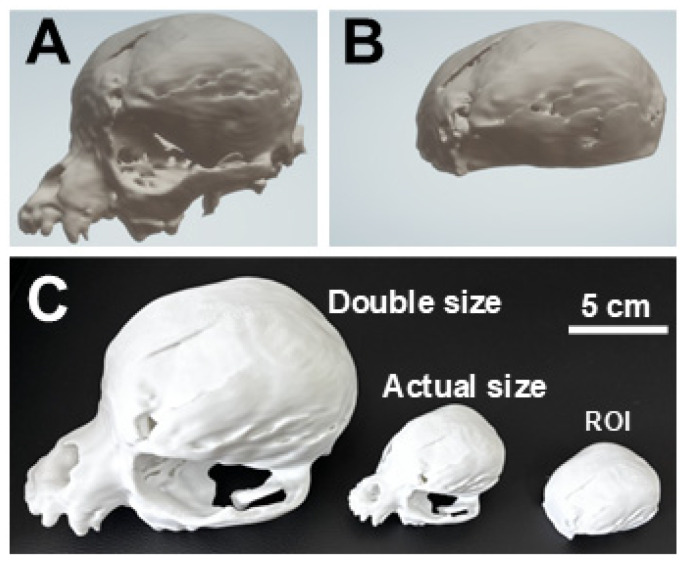
Comparative visualization of digital and physical 3D models. (**A**) Upper skull digital model. (**B**) Region of Interest (ROI) digital model. (**C**) Corresponding 3D-printed physical models.

Two 3D printable models were prepared based on the 0.5^3^ mm^3^ resolution data: the upper skull (Figure 5A) and a ROI model focusing on the frontal fracture site (Figure 5B). The upper cranial vault model was created by first disconnecting the mandible using the “Erase” tool, followed by using the “Keep selected island” function to retain only the main skull segment. This process took approximately 5 min. For the ROI model, the internal voids were filled manually using the “Paint” tool to create a solid structure, and all regions outside the ROI were removed using the “Erase” tool. The base of the ROI model was flattened to ensure the fracture region faced upward during printing.

The resulting physical 3D prints are shown in Figure 5C. Fused Deposition Modeling (FDM) with thermoplastic was employed due to its cost-effectiveness and broad accessibility. Polylactic acid (PLA) was selected for its biocompatibility and widespread use in orthopedic applications [15]. The upper cranial vault model was printed at both actual size and double size, while the ROI model was printed at actual size only. 3D printing was performed by a randomly selected vendor on Taobao.com, with the printed model delivered four days after order placement. Detailed printing parameters and associated costs are summarized in Table 1.

The reader-based assessment of 2D CT and 3D visualization showed that the composite clinical utility score increased from a median of 25 (IQR 23–26) in the 2D round to 30 (IQR 28–30) in the 3D round. The median paired increase was 3 points (IQR, 2–7) on a 30-point scale. In an exploratory paired Wilcoxon signed-rank test, this increase showed a positive trend (*p* = 0.063). Among the six individual Likert-scale items, the greatest improvements were observed for spatial anatomical understanding, surgical planning value, and overall clinical usefulness, which increased from 4 (IQR, 3–4) to 5 (IQR, 5–5), from 4 (IQR, 3–4) to 5 (IQR, 5–5), and from 4 (IQR, 3–5) to 5 (IQR, 5–5), respectively. Fracture-line visibility and fracture morphology understanding were already rated highly in the 2D round (5 scores). For the 3D-specific assessment, the average score was 27.8/30, indicating a strong perceived added value of 3D visualization. Evaluators also recognized the potential risk of relying on 3D visualization without reference to the original CT images (3.8/5). All evaluators rated the 3D materials as providing significant added value compared with 2D CT alone.

## 4. Discussion

This study provides a practical, step-by-step guide to achieving three key objectives from raw CT DICOM data using an open-source platform: (1) rapid 3D visualization of bone surface models, (2) voxel resampling for surface visualization, and (3) preparation of clean, printable models for 3D printing [4]. The workflow is efficient (requiring approximately 1, 5, and 20 min for each respective task), cost-effective (utilizing free software and low-cost 3D printing with material costs below 1 USD per model), and practical, although basic familiarity with DICOM import, bone-window anatomy, segmentation tools, and STL export remains necessary [11]. In our case, printing a real-size upper cranial vault model required approximately 40 g of plastic filament (costing about 0.15 USD in China) and roughly 3 h of print time. In addition, because the model was reconstructed from a routine clinical CT dataset, common CT-related factors such as partial-volume averaging, image noise, beam-hardening artifacts, field of view, and reconstruction settings may affect the clarity of small cranial bone surface models. The workflow steps are presented to support reproducibility, while the practical value lies in improving understanding of fracture morphology, spatial relationships, and potential neurosurgical or craniofacial planning considerations.

Compared to proprietary software bundled with CT scanners, this method offers a convenient, customizable, and affordable alternative, particularly suitable for small animal cases and time-sensitive scenarios such as acute traumatic injuries [16]. In this retrospective technical demonstration for a complex cranial fracture in a Yorkshire Terrier, the 3D materials were used to assess perceived visualization and planning value [17].

The exploratory reader-based assessment provides preliminary qualitative information on the practical visualization value of the proposed workflow. Although all evaluators were able to identify the cranial fracture using the 2D CT materials alone, the 3D rounds were rated more favorably for perceived usefulness. Importantly, these findings should not be interpreted as evidence that 3D reconstruction increases the intrinsic spatial resolution or diagnostic sensitivity of the original CT data; rather, they support its role as an adjunctive visualization tool for improving understanding of complex fracture morphology and facilitating clinical communication. This interpretation is consistent with recent veterinary studies showing that CT-derived 3D models can support presurgical planning, client communication, and intraoperative guidance [18] and improve fracture alignment and reduce surgical time in canine radial fractures [8].

**Figure 6:**
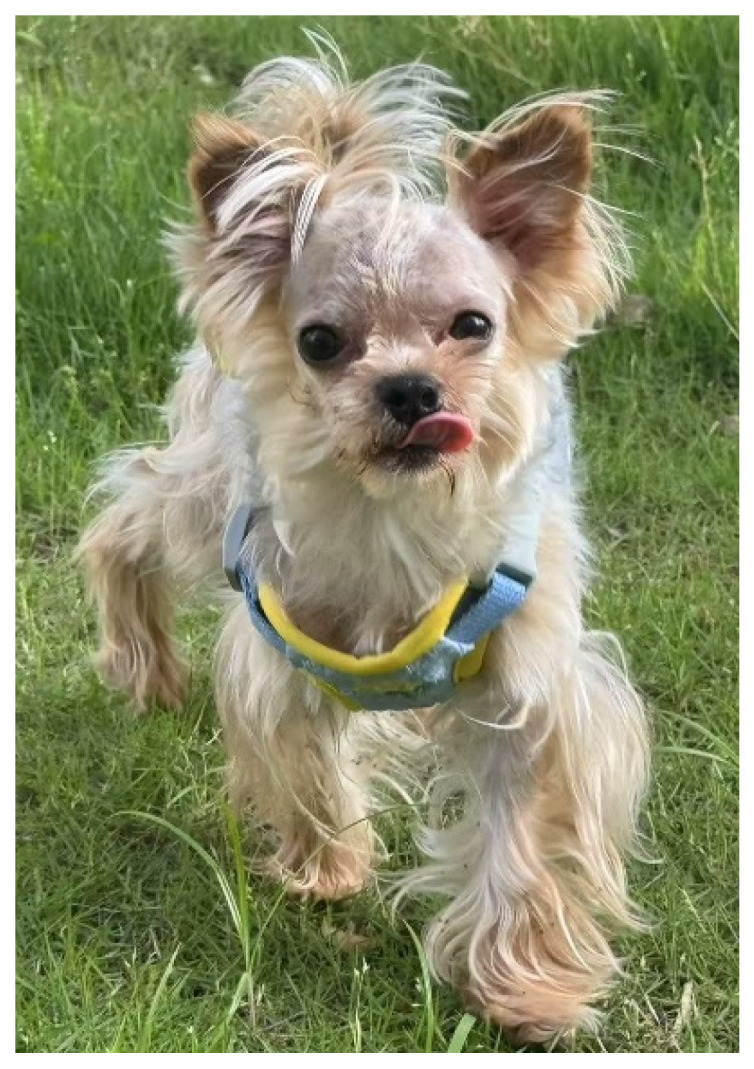
Follow-up photograph of the patient on the day of hospital discharge, 22 days after the accident.

Beyond its academic contribution, this case also became a widely shared social event. The rescue effort garnered significant public attention, with over 30 social media posts and more than 17 million online views. It mobilized support from over 500 individual donors, none of whom knew each other personally, reflecting broad public engagement in animal welfare. The dog successfully underwent surgery and was discharged from the hospital 22 days after the accident (Figure 6). We are proud that the academic community played a role in this story and hope this report raises further awareness of the need for companion animal protection in our society.

This study has several limitations. First, 3D Slicer is an open-source research and educational platform and should be used as an adjunctive visualization tool rather than as a certified standalone diagnostic or surgical-planning device; clinical decisions should be made by qualified clinicians using the original CT data and applicable regulatory standards. Second, although we used 3D Slicer due to its popularity, active user community, and rich tutorial base, other free tools such as Drishti (github.com/nci/drishti, accessed on 4 May 2025) and ITK-SNAP (www.itksnap.org, accessed on 4 May 2025) were not evaluated. Third, the workflow was carried out by a single experienced orthopedic engineer specializing in human imaging; reproduction by veterinary users with different levels of experience remains to be validated. Operators should have basic familiarity with DICOM import, CT bone-window anatomy, threshold-based segmentation, segment editing tools such as Island/Erase/Paint, and STL export. Fourth, the clinical utility and outcome impact of the 3D models were not formally assessed. Due to the urgency of the rescue, we were only able to conduct a retrospective demonstration of the method. Nonetheless, this case may help illustrate the feasibility of low-cost CT-derived 3D visualization in veterinary care and inform future controlled studies in more suitable clinical contexts [19]. Fifth, the CT protocol was part of routine trauma imaging performed at the treating hospital and was not specifically optimized for detailed cranial evaluation. Key acquisition parameters could not be retrospectively verified from the exported DICOM metadata. Because repeat imaging was not ethically or clinically justified in this recovered patient, the acquisition protocol could not be retrospectively optimized. Therefore, the findings should be interpreted as a technical demonstration based on available routine clinical imaging rather than as evidence of optimized cranial CT performance or improved diagnostic accuracy. In addition, formal STL-to-print dimensional validation was not performed in this single retrospective case; controlled veterinary evidence indicates that 3D-printing accuracy is affected by bone size and printer type, and therefore requires a dedicated validation design [20].

## 5. Conclusions

This technical demonstration supports the feasibility of a low-cost, open-source CT-to-3D workflow for small-animal cranial trauma; its main value is adjunctive visualization and communication, and future prospective studies should determine whether it improves surgical planning, operative efficiency, or patient outcomes. This technical demonstration shows that routine veterinary CT data can be converted into 3D digital and physical skeletal models using an open-source, low-cost workflow in approximately 20 min and printed in 0.7 h (approximately 40 min for the ROI model), whereas the actual-size upper cranial vault model was prepared in approximately 5 min and printed in 2.9 h. These printing times are reported as case-specific examples and may vary with slicing software, printer model, orientation, layer thickness, infill, support settings, and other printing parameters. The upper cranial vault model served as an effective tool for surgical planning efficiency and anatomical visualization. Material costs were under 1 USD per model. This workflow offers a feasible and low-cost adjunct for CT-derived 3D visualization and printable model generation in small-animal cranial trauma, but larger prospective studies are required to determine its effect on diagnostic accuracy, surgical planning, operative performance, and outcomes.

## Figures and Tables

**Table 1:** 3D Printing Parameters and Costs.

Parameters		Values		
Service cost total */CNY		56		
Service time consumption **/days		4		
Machine		Bamboo Lab P1P		
Material		CAILAB white PLA		
Layer thickness/mm		0.2		
	**Upper skull (double size)**		**Upper skull** **(actual size)**	**Fractured region** **(actual size)**
Material consumption/g	208.7		42.4	19.1
Material cost/CNY	5.2		1.1	0.5
Time consumption/hour	11.1		2.9	0.7

* Service cost total is the total amount payable to the vendor, including the material, service, vendor handling and shipping cost. ** Service time refers to the vendor-dependent fabrication and delivery time in calendar days from the selected Taobao.com, accessed on 4 May 2025, printing service and may vary across vendors, locations, and service conditions.

## Data Availability

The data presented in this study are openly available in Figshare at https://doi.org/10.6084/m9.figshare.29039192.v1. The dataset includes de-identified CT and MRI scans from a 2-year-old male Yorkshire Terrier with cranial trauma. The DICOM imaging data were de-identified using the RSNA DICOM Anonymizer, version 17.4.6, Radiological Society of North America. The dataset is available for academic and non-commercial use under the CC BY 4.0 license.

## Supplementary Materials

The following supporting information can be downloaded at: https://www.mdpi.com/article/10.3390/vetsci13070610/s1, Supplementary File S1: Reader-Based Assessment Form for CT and 3D Visualization in Small-Animal Cranial Trauma.
