# Supplementary material for: Practical 3D Reconstruction and 3D Printing of Veterinary CT Scans in Small Animals: A Technical Demonstration with Reader-Based Validation in Canine Cranial Trauma
Source: Vet Sci. 2026 Jun 24;13(7):610. doi: 10.3390/vetsci13070610 (PMC13431402; doi:10.3390/vetsci13070610)
Supplement: Supplementary file 1 [file vetsci-13-00610-s001.zip › vetsci-4288952 Supplementary File S1.pdf]

Supplementary File S1

Reader-Based Assessment Form for CT and 3D Visualization in Small-Animal Cranial Trauma

Consent and data-use statement. By submitting this questionnaire, you confirm that your responses are truthful and objective, and you agree that the anonymized questionnaire data may be used for scientific analysis and publication. This questionnaire does not collect personally identifiable information. We sincerely thank you for your support and contribution to this study.

I. Evaluator Information

1. Professional background

☐ Veterinary imaging
☐ Medical imaging
☐ Medical engineering / 3D reconstruction
☐ Veterinary surgery / orthopaedics / neurosurgery
☐ Orthopaedics / neurosurgery
☐ Other: ____________________

2. Years of relevant experience

☐ <3 years ☐ 3-5 years ☐ 5-10 years ☐ >10 years

II. Case Vignette

An approximately 2-year-old male Yorkshire Terrier weighing 2.2 kg underwent CT examination after traffic-related cranial trauma. Please independently assess the cranial osseous injury based on the imaging materials provided. The original radiology report, operative records, intraoperative findings, and study conclusions are not provided.

III. Objective Assessment

Please answer the following questions based on the imaging materials provided in the current round.

1. Is a cranial fracture present?

☐ Yes ☐ No ☐ Uncertain

2. Which anatomical regions are mainly involved? Multiple selections are allowed.

☐ Frontal bone region
☐ Occipital bone region
☐ Nasal bone / facial skeleton region
☐ Other: ____________________
☐ Parietal bone region
☐ Orbital rim / periorbital structures
☐ Unable to determine

3. Is there a depressed fracture?

☐ Yes ☐ No ☐ Uncertain

4. Is there an obvious displaced or free bone fragment?

☐ Yes ☐ No ☐ Uncertain

5. Is there possible involvement of the orbital rim or adjacent craniofacial structures?

☐ Yes ☐ No ☐ Uncertain

IV. Five-Point Rating Items

Please rate each item based on the imaging materials provided in the current round.

Assessment item	Rating
Fracture-line visibility: I can clearly identify or trace the main fracture line.	☐ 1 ☐ 2 ☐ 3 ☐ 4 ☐ 5
Understanding of fracture morphology: I can understand whether the fracture is depressed,	☐ 1 ☐ 2 ☐ 3 ☐ 4 ☐ 5

Assessment item	Rating
displaced, comminuted, or linear.	
Spatial anatomical understanding: I can understand the three-dimensional relationship between the fracture and the orbital rim, cranial vault, and adjacent osseous structures.	☐ 1 ☐ 2 ☐ 3 ☐ 4 ☐ 5
Diagnostic confidence: I am confident in my assessment of fracture location and morphology.	☐ 1 ☐ 2 ☐ 3 ☐ 4 ☐ 5
Surgical-planning value: The imaging information helps assess surgical exposure, bone-fragment management, and preoperative communication.	☐ 1 ☐ 2 ☐ 3 ☐ 4 ☐ 5
Overall clinical usefulness: The imaging materials provide practical value for understanding this case.	☐ 1 ☐ 2 ☐ 3 ☐ 4 ☐ 5

Rating scale: 1 = very poor / very uncertain; 2 = poor; 3 = fair; 4 = good; 5 = excellent / highly confident.

V. Clinical Decision-Making and Additional Information

1. Based on the current imaging materials, are these data sufficient to support preliminary surgical planning?

☐ Yes ☐ No ☐ Uncertain

2. What additional information would be helpful for surgical decision-making? Multiple selections are allowed.

☐ Complete original DICOM dataset
☐ Higher-resolution CT or optimized CT acquisition parameters
☐ MRI information on soft tissue / brain injury
☐ Preoperative neurological assessment
☐ Intraoperative findings
☐ 3D digital model
☐ Physical 3D-printed model
☐ None
☐ Other: ____________________

VI. 3D Round Only: Added Value of 3D Visualization Compared with 2D CT Alone

Please evaluate the added value of the 3D visualization materials compared with 2D CT alone.

Assessment item	Rating
The 3D model makes the fracture region more intuitive to understand.	☐ 1 ☐ 2 ☐ 3 ☐ 4 ☐ 5
The 3D model helps me understand the course of the fracture line and the spatial position of bone fragments.	☐ 1 ☐ 2 ☐ 3 ☐ 4 ☐ 5
The 3D model improves my understanding of surgical exposure or bone-fragment management.	☐ 1 ☐ 2 ☐ 3 ☐ 4 ☐ 5
The 3D model would help explain the injury to the surgical team or animal custodian.	☐ 1 ☐ 2 ☐ 3 ☐ 4 ☐ 5
Without replacing interpretation of the original CT images, the 3D model can serve as a valuable adjunctive tool.	☐ 1 ☐ 2 ☐ 3 ☐ 4 ☐ 5
If used alone without reference to the original CT images, the 3D model may carry a risk of misleading interpretation.	☐ 1 ☐ 2 ☐ 3 ☐ 4 ☐ 5

Rating scale: 1 = strongly disagree; 2 = disagree; 3 = neutral; 4 = agree; 5 = strongly agree.

VII. 3D Round Only: Overall Evaluation

1. Compared with 2D CT alone, the additional value of the 3D visualization materials was:

☐ No obvious added value
☐ Minor added value
☐ Moderate added value
☐ Significant added value

2. What was the most helpful aspect of the 3D materials?

____

____

3. What was the main limitation of the 3D materials?

____

____

Note. *This Supplementary File presents an English translation of the original Chinese questionnaire used for the reader-based assessment.*
